# Autophagy lessens ischemic liver injury by reducing oxidative damage

**DOI:** 10.1186/2045-3701-3-26

**Published:** 2013-06-10

**Authors:** Kai Sun, Xuqin Xie, Yan Liu, Zhipeng Han, Xue Zhao, Ning Cai, Shanshan Zhang, Jianrui Song, Lixin Wei

**Affiliations:** 1 Medical Sciences Research Center, Renji hospital, School of Medicine, Shanghai Jiaotong University, Shanghai, China; 2 Tumor Immunology and Gene Therapy Center, Eastern Hepatobiliary Surgery Hospital, The Second Military Medical University, Shanghai, China; 3 Department of Radiation Oncology, Affiliated Tumor Hospital of GuangXi Medical University, Guangxi, China

**Keywords:** Liver ischemia, Nutrient deprivation, Autophagy, Reactive oxygen species, Necrosis

## Abstract

**Background:**

Hepatic ischemia/reperfusion is a multi-factorial process which causes liver injury. It is reported that ischemia alone is sufficient to induce liver injury. Nutrient deprivation is a crucial factor impacting ischemic injury of the liver. Therefore, we explored the role of autophagy in ischemia through using hepatic ischemia rat model *in vivo* and nutrient-free model *in vitro*.

**Results:**

We found that both ischemia *in vivo* and nutrient deprivation *in vitro* activated autophagy, inhibition of which aggravated ischemia- or nutrient deficiency-induced injury. In the nutrient-free condition, autophagy inhibition enhanced liver cell necrosis but not apoptosis by promoting reactive oxygen species (ROS) accumulation, and antioxidant NAC could reverse this trend. Inhibition of autophagy also resulted in the increase of the percentage of necrotic cell but not apoptotic cell in the ischemia-treated rat livers. Further studies showed that under nutrient deprivation, autophagy inhibition promoted mitochondrial ROS generation, which further aggravated mitochondria damage. These changes formed a “vicious cycle” that accelerated the process of cell necrosis. Autophagy inhibition also increased mitochondrial oxidative stress during hepatic ischemia, and antioxidant could suppress the aggravation of ischemia-induced liver damage in the co-treatment of autophagy inhibitor.

**Conclusions:**

Taken together, our results suggested that autophagy suppressed ischemic liver injury by reducing ROS-induced necrosis. This finding will contribute to the development of the therapeutic strategy about the pre-treatment of liver surgery.

## Introduction

Hepatic ischemia/reperfusion (I/R) is an important causing liver injury during liver surgery, especially in hepatic transplantation, hepatic resection, and trauma. I/R injury has a profound impact on the burden of liver diseases. However, how to improve liver function in the process of I/R is always a challenge due to incomplete understanding of the mechanism of I/R injury. Although the studies about I/R are nearly all focused on reperfusion, long time ischemia is also a crucial damage factor in liver injury. Understanding the mechanism of ischemia damage is important for reducing liver injury during surgery. Interruption of an organ’s blood flow subsequently leads to its lack of oxygen and nutrient supply, loss of ATP, and acidosis. Among the consequences, nutrient deprivation is a very important factor impacting liver ischemic injury [1]. Macroautophagy (hereafter referred as autophagy) may play a crucial role in response to nutrient deprivation.

Autophagy is an evolutionary conserved process involved in degradation of long-lived proteins and excess or dysfunctional organelles [2]. During the process of autophagy, cellular contents including organelles are sequestered in double-membrane vesicles called autophagosomes, then the autophagosomes fuse with lysosomes where hydrolysis or cargo occurs, supplying amino acids and macromolecular precursor for cells [2,3]. Autophagy occurs at low levels under normal conditions and is important for the turnover of organelles [4,5].

In recent years, various studies reported that autophagy could promote survival in response to ischemia. Wang, P. found that induction of autophagy contributed to the neuro-protection of nicotinamide phosphoribosyltransferase in cerebral ischemia [6]. Hoshino, A. showed that p53-TIGAR axis attenuated mitophagy to exacerbate cardiac damage after ischemia [7]. However, the mechanism by which autophagy protects cells from ischemia injury has not been clarified.

In our study we investigated the effect of autophagy on survival of hepatocytes in hepatic ischemia. We reported here that both ischemia *in vivo* and nutrient deprivation *in vitro* significantly induced autophagy. Inhibition of autophagy aggravated ischemia-induced liver injury and starvation-induced hepatocyte death. Notably, these increased cell death was mainly due to necrosis but not apoptosis. Further study showed that inhibition of autophagy aggravated starvation-induced reactive oxygen species (ROS) accumulation, especially mitochondrial ROS, which in turn led to further mitochondria damage. These excessive ROS contributed to hepatocyte necrosis. Meanwhile, autophagy inhibition also enhanced mitochondrial oxidative stress in the process of hepatic ischemia, thus resulted in aggravated liver injury, which could be significantly suppressed by antioxidant.

## Results

### Autophagy protects liver from ischemic injury in the rats

**Figure 1:**
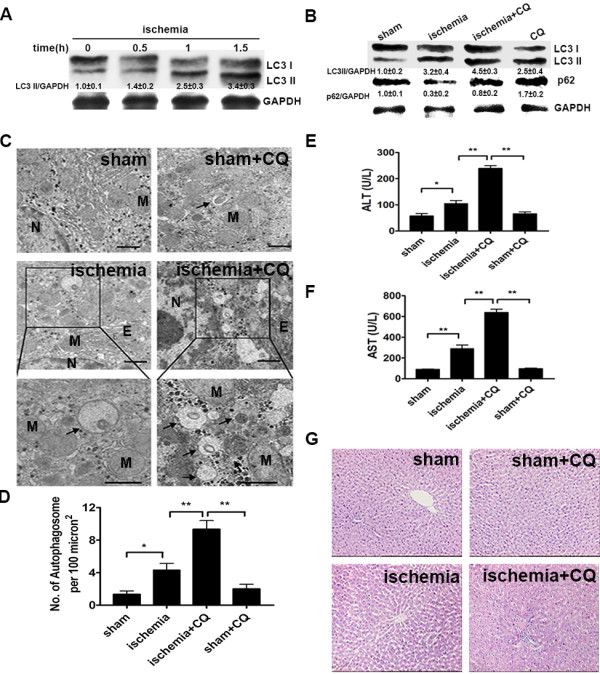
**Inhibition of autophagy accelerated ischemia-induced liver injury.** (**A**) The rats were treated as the indicated ischemic time, and then their liver fractions were analyzed by immunoblot assay. Semiquantitative densitometry analysis (versus GAPDH) of LC3 II was performed for each sample. Data were shown as mean ± SEM (n = 3). (**B**) Four groups of rats were treated as indicated. After 90 min of ischemia treatment, the protein levels of LC3 and p62 in their liver sections were analyzed by immunoblot assay. Data were shown as mean ± SEM (n = 3). (**C**) The liver samples were processed for EM. And representative electron micrographs were shown. The photos at the bottom panel were high magnification of electron micrographs. Arrows denote autophagic vacuoles (N: nucleus; M: mitochondria; Bar: 1 μm). (**D**) The number of autophagosome per 100 μm^2^ in the electron micrographs was determined. Data were shown as mean ± SEM (n = 6; *: p < 0.05; **: p < 0.01). (**E** and **F**) The serum ALT (**E**) and AST (**F**) levels of rats from the indicated groups were detected. Data were shown as mean ± SEM (n = 6; *: p < 0.05; **: p < 0.01). (**G**) Liver samples of the indicated groups were processed for H&E. Representative images were shown with an original magnification × 200.

Long time ischemia alone is able to cause great damage during hepatic surgery. Since autophagy is generally considered as a protective mechanism in response to stresses, we detected the impact of ischemia on autophagy level of liver cells in the rats, and used autophagy inhibitor chloroquine (CQ) to determine whether autophagy protects hepatocytes from ischemia damage. The inhibition of autophagic flux by CQ can cause the accumulations of autophagosome, microtubule-associated protein 1 light chain 3 (LC3) II, and p62/SQSTM1, which is an ubiquitin-binding scaffold protein selectively degraded by autophagy [8,9]. Immunoblot analysis suggested that the level of LC3 II was increased after liver ischemia treatment (Figure 1A). Co-treatment of rats with CQ further increased the level of LC3-II but decreased the degradation of p62 (Figure 1B). Electron microscope (EM) analysis also showed that autophagosome was rarely detected in the livers of sham group, but an increased number of autophagosomes were detected 90 minutes after ischemic surgery. Meanwhile, compared to ischemia group, ischemia + CQ group had marked ultrastructural alterations in the livers, such as autophagosome accumulation, swollen mitochondria and disordered endoplasmic reticulum (ER) (Figure 1C and D). These results suggested that autophagy was activated in ischemic hepatocytes. And CQ inhibited this autophagic flux and caused cellular structural abnormality in ischemic hepatocytes.

Then we examined the impact of autophagy inhibition on ischemia-induced liver injury. The serum levels of alanine aminotransferase (ALT) and aspartate aminotransferase (AST), two classical markers of liver injury, were prominently increased in the ischemia-treated rats as compared to those of normal rats. CQ injection before ischemia led to two fold or more increase in the levels of ALT and AST, but CQ treatment alone had no obvious influence on the levels of ALT and AST (Figure 1E and F). The hematoxylin and eosin (H&E) staining also revealed that ischemia group had significant liver injury such as loosing hepatocyte cords and ischemia + CQ group had further marked morphological alterations in the livers, including unclear structure of hepatic lobules, disarranged hepatocyte cords, narrowed hepatic sinusoids and swollen hepatocytes (Figure 1G). These data demonstrated that inhibition of autophagy significantly increased liver injury during ischemia.

### Autophagy protected liver cells from cell necrosis induced by nutrient deprivation

**Figure 2:**
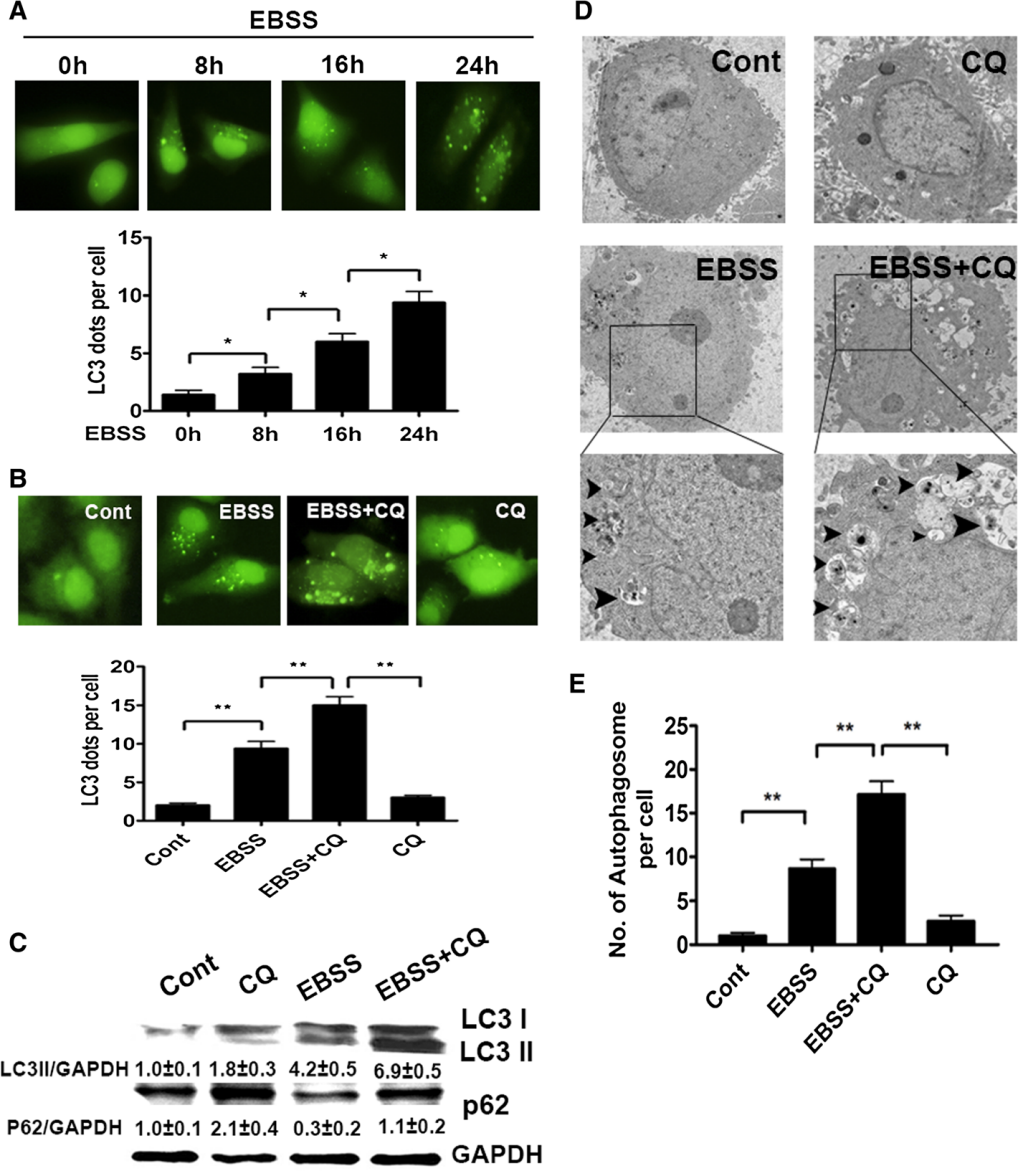
**Nutrient deprivation activated autophagy in liver cells *in vitro*.** (**A, B**) Chang liver cells were transfected with GFP-tagged LC3. After 24 hours transfection, cells were incubated in EBSS for indicated times (**A**), or were cultured in indicated conditions for 24 h (**B**). Then the dots were observed under a fluorescent microscope. Representative images of treated cells were shown with an original magnification × 400. GFP-LC3 dots per cell were counted for quantification. Data were shown as mean ± SEM (n = 200; *: p < 0.05; **: p < 0.01). (**C**) Chang liver cells were cultured in indicated conditions for 24 h. Whole cell lysates were subjected to western blot to detect the indicated antibodies. (**D**) Chang liver cells incubated in the indicated conditions for 24 h were processed by EM. Black triangles indicate the autophagosome (Bar: 1 μm). (**E**) Quantification of the number of autophagosome per cell. Data were shown as mean ± SEM (n = 10; **: p < 0.01).

Nutrient deprivation is a major factor in ischemia-induced liver injury [1]. To explore how autophagy protected liver cells from ischemia, we treated Chang liver cells with Earle’s balanced salt solution (EBSS) to mimic ischemia induced-nutrient deprivation. Firstly, we used a GFP-LC3 reporter to examine the influence of nutrient deprivation on autophagy level of liver cells. GFP-LC3 dot is an indicator of autophagosome formation. The result showed that EBSS treatment increased GFP-LC3 dots in the Chang liver cells in a time-dependent manner (Figure 2A). Furthermore, CQ treatment caused more GFP-LC3 dots accumulation in the nutrient-free condition (Figure 2B). Meanwhile, we used immunoblot to detect the levels of LC3II and p62. LC3 II level was increased in the nutrient-free condition while p62 level was decreased. Compared to EBSS treatment alone, co-treatment of EBSS and CQ led to further accumulation of LC3-II, and retarded p62 degradation (Figure 2C). EM analysis also revealed an obvious gathering of autophagosomes following nutrient-free treatment, and CQ treatment resulted in more autophagosomes accumulation (Figure 2D and E). These results suggested that nutrient deprivation *in vitro* induced autophagy in Chang liver cells and this autophagic flux could be inhibited by CQ treatment.

**Figure 3:**
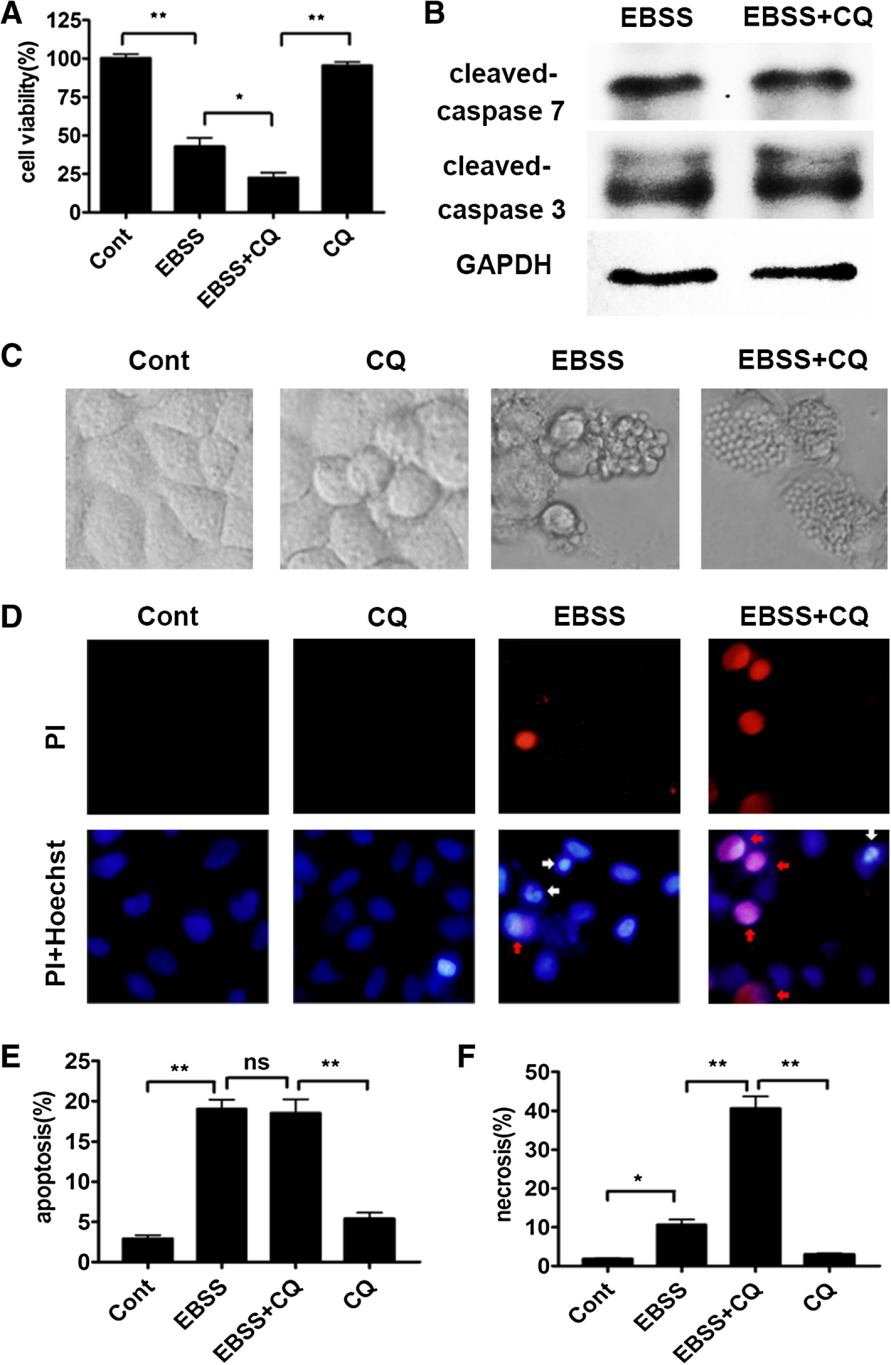
**Autophagy inhibition aggravated Chang liver cell necrosis in response to nutrient deprivation.** Chang liver cells were cultured in complete medium or EBSS for 24 h in the absence or presence of 10 μM CQ. (**A**) Cell viability was measured by CCK8. Data were shown as mean ± SEM (n = 6; *: p < 0.05; **: p < 0.01). (**B**) Whole cell lysates of the EBSS and EBSS + CQ groups were subjected to detect the indicated antibodies by immunoblot assay. (**C**) The morphologies of cells were captured by a light microscope. (**D**, **E** and **F**) The representative images of Hoechst 33342/PI staining were shown at magnification × 400 (**D**). PI positive/ Hoechst strong positive cells with condensed nuclei were considered as apoptotic ones (white arrows), and PI strong positive/ Hoechst weak positive cells were regarded as necrotic ones (red arrows). The percentage of apoptosis (**E**) or necrosis (**F**) was shown. Data were shown as mean ± SEM (n = 3; *: p < 0.05; **: p < 0.01; ns: no significance).

Then we examined the impact of autophagy on the survival of liver cells under nutrient deficiency. Cell Counting Kit-8 (CCK8) assay showed that the EBSS + CQ group had less cell viability than the EBSS group (Figure 3A). However, the levels of apoptosis-associated proteins, cleaved-caspase7 and cleaved-caspase3, had no significant difference between the EBSS and EBSS + CQ groups (Figure 3B). Severe shrinkage and rounding of highly refringent cells, the main morphological features of apoptosis, were observed in the EBSS group. Nevertheless, the EBSS + CQ group had not only apoptotic cells but also the swell and flattened cells with vast membrane bubbles, which were the typical necrotic cells (Figure 3C) [10]. Hoechst 33342/PI staining analysis also showed that CQ had not significantly impacted on the apoptotic level of liver cells in the nutrient-free condition, but led to the prominent increase of liver cell necrosis (Figure 3D, E and F).

**Figure 4:**
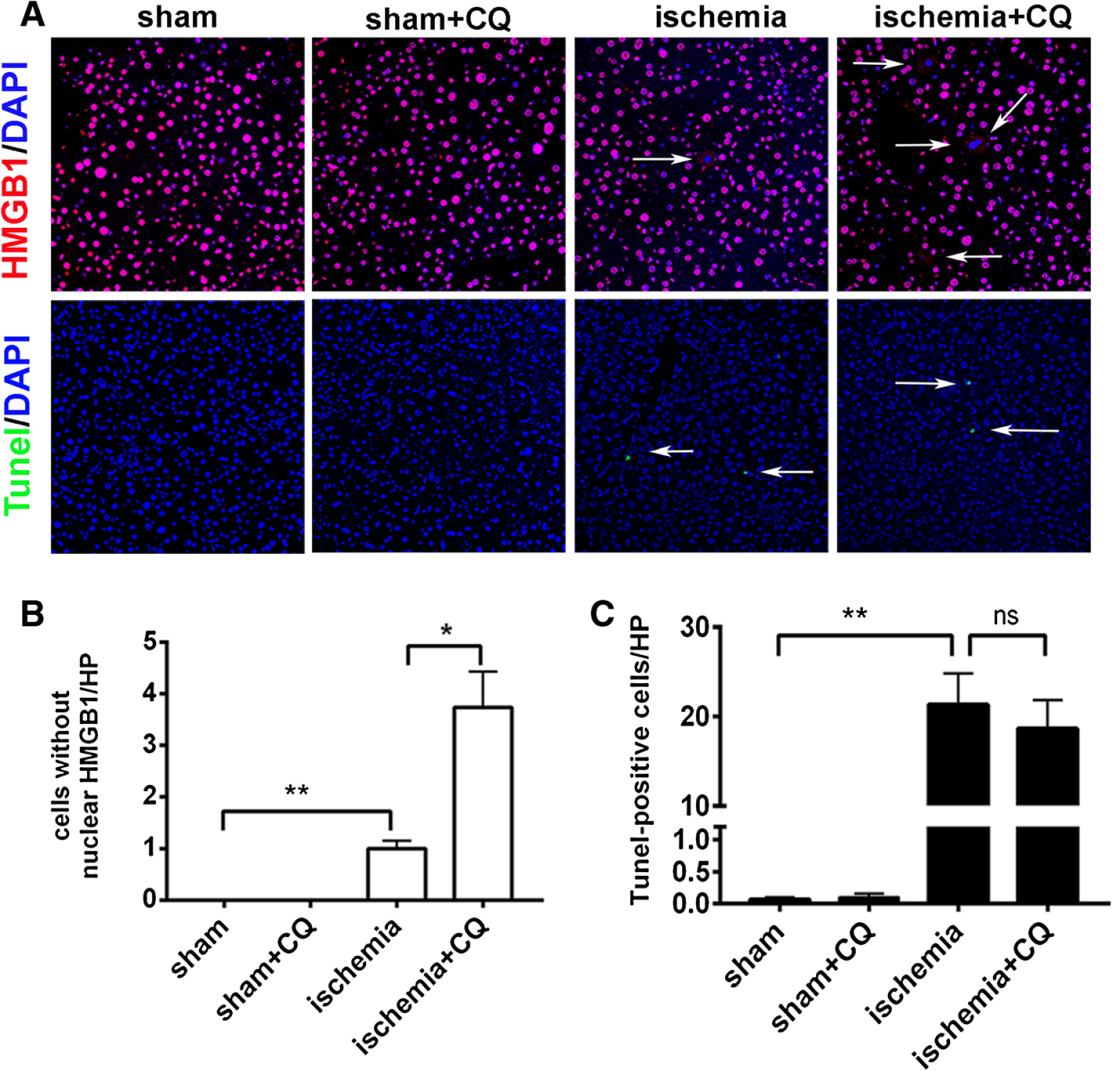
**Autophagy inhibition increased ischemia-induced cell necrosis but not apoptosis in the rat livers.** (**A**) Four groups of rats were treated as indicated. Then their liver sections were detected by immunohistofluorescence staining of HMGB1 (upper) and Tunel staining (lower). Arrow indicated the cells with the release of HMGB1 from the nuclei (upper) and Tunel-positive cells (lower). (**B** and **C**) The number of cells without nuclear HMGB1 (**B**) and Tunel-positive cells (**C**) per high power field (HP, magnification × 400) were shown in the graph. Data were shown as mean ± SEM (n = 5; *: p < 0.05; **: p < 0.01; ns: no significant difference).

We further detected whether autophagy inhibition aggravated liver cells necrosis but not apoptosis during ischemia treatment. HMGB1, a nucleoprotein which usually binds to chromatin, is released from the nuclei of necrotic cell but not apoptotic cell [11]. In the hepatocytes of rats of the sham and CQ groups, HMGB1 was solely located in the nuclei. However, HMGB1 revealed a cytosolic pattern in ischemia-treated hepatocytes, which indicated that ischemia-induced HMGB1 release. Moreover, the rats of the ischemia + CQ group had higher percentage of cells with the release of HMGB1 from the nuclei compared to that of the rats of the ischemia group (Figure 4A and B). Meanwhile, co-treatment of CQ did not lead to the increase of apoptotic (Tunel-positive) hepatocytes in the ischemia-treated rats (Figure 4A and C).

Taken together, these data suggested that autophagy protected liver cells against nutrient deprivation-induced cell necrosis.

### Autophagy protected liver cells from nutrient deprivation-induced necrosis by eliminating ROS-generating mitochondria *in vitro*

**Figure 5:**
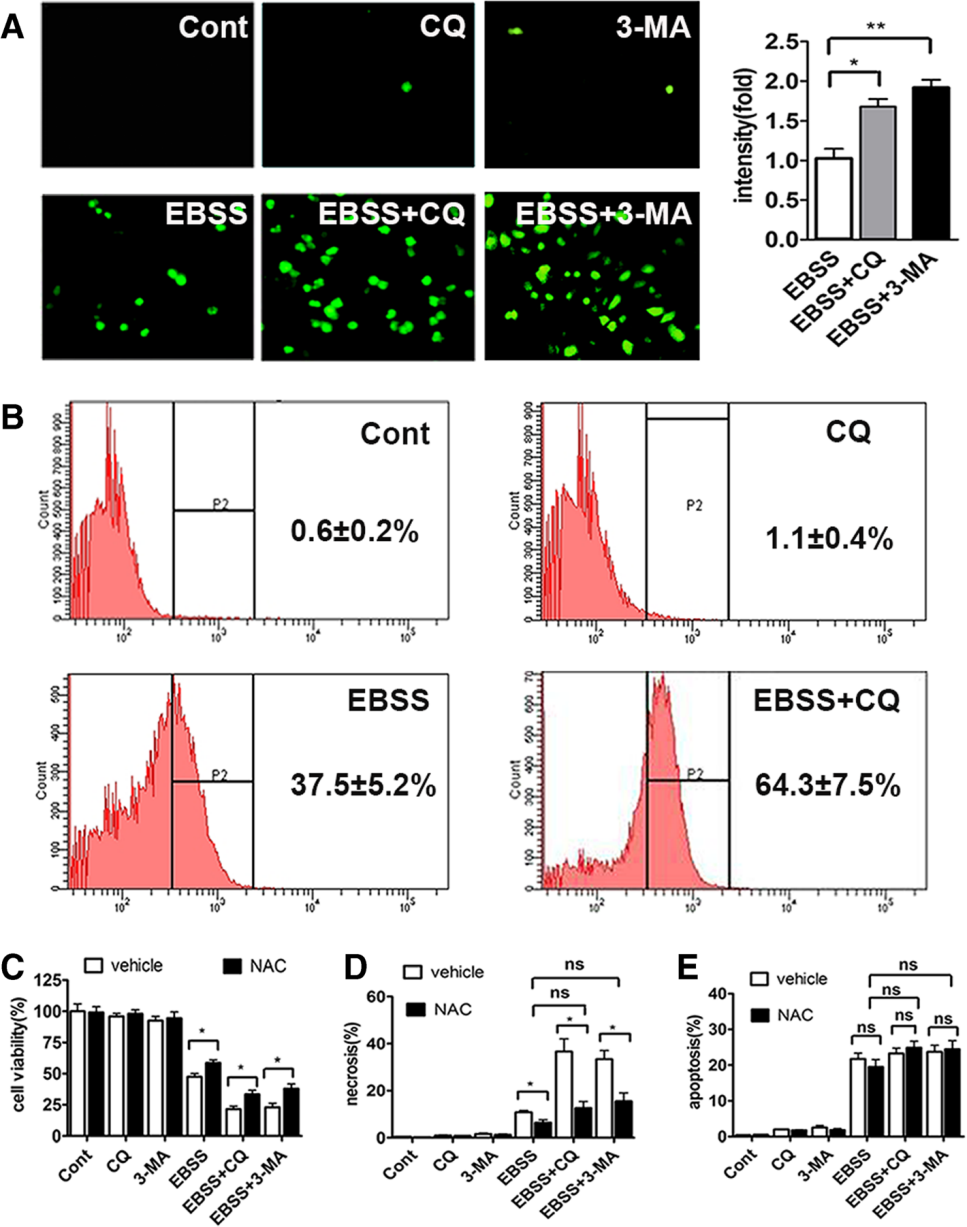
**Inhibition of autophagy accelerated nutrient deprivation-induced ROS accumulation and subsequent cell necrosis.** (**A**) Chang liver cells were cultured with complete medium or EBSS in the absence or presence of CQ or 3-MA for 12 h. At the end of treatments, cells were stained with DCF-DA and observed under a fluorescent microscope at magnification × 400 (left panel). The fluorescent intensity of each group was also quantified (right panel) and expressed as mean ± SEM (n = 3; *: p < 0.05; **: p < 0.01). (**B**) Chang liver cells were cultured with complete medium or EBSS in the absence or presence of CQ or 3-MA for 24 h. Intracellular ROS generation was measured by flow cytometry using DCF-DA staining. ROS positive cells were counted and expressed as the mean ± SEM (n = 3). (**C**, **D** and **E**) Chang liver cells were cultured with complete medium or EBSS in the absence or presence of CQ or 3-MA and/or NAC for 24 h. Cell viability was detected by CCK8, and necrosis and apoptosis of Chang liver cells were assayed by Hoechst 33342/PI staining. Cell viability (**C**), the percentage of necrosis (**D**) and apoptosis (**E**) were shown. Data were shown as mean ± SEM (n = 3; *: p < 0.05; ns: no significance).

Many pathogenic and physical processes including ischemia connect with ROS. Moreover, many studies reported that autophagy had interaction with ROS [12,13]. So we hypothesized that autophagy may protect liver cells from nutrient deprivation-induced necrosis by eliminating oxidative stress. Compared to the cells in nutrient-rich condition, there was a marked generation of ROS in those cells under nutrient-free condition. Co-treatment of EBSS with CQ further enhanced ROS generation. Then to confirm this observation, we used another autophagy inhibitor 3-Methyladenine (3-MA) and obtained a similar result (Figure 5A). Consistent with the above results, flow cytometry assay showed that the percentage of DCF positive cell was just 0.6% in control group, while it ran up to 37.5% in the EBSS group. In the EBSS + CQ group, it was much higher, reaching about 64.3% (Figure 5B). These data indicated that starvation-treated hepatocytes had significant ROS accumulation, which was further aggravated by autophagy inhibition.

To examine whether autophagy played its protective role mainly through eliminating oxidative stress in the nutrient-free condition, we used antioxidant N-acetyl-L-cysteine (NAC) to treat Chang liver cells. Examination of cell viability showed that NAC prominently promoted the survival of Chang liver cells in the EBSS, EBSS + CQ and EBSS + 3-MA group (Figure 5C). Hoechst 33342/PI staining assay also suggested that NAC significantly attenuated cell necrosis in the EBSS and EBSS + CQ groups. More importantly, the level of cell necrosis had no obvious difference between EBSS, EBSS + CQ and EBSS + 3-MA group after NAC treatment (Figure 5D). Notably, NAC treatment had no influence on the apoptotic level of cells in the EBSS, EBSS + CQ and EBSS + 3-MA groups (Figure 5E). These results demonstrated that autophagy protected Chang liver cells from nutrient deprivation-induced cell necrosis by eliminating excessive ROS.

**Figure 6:**
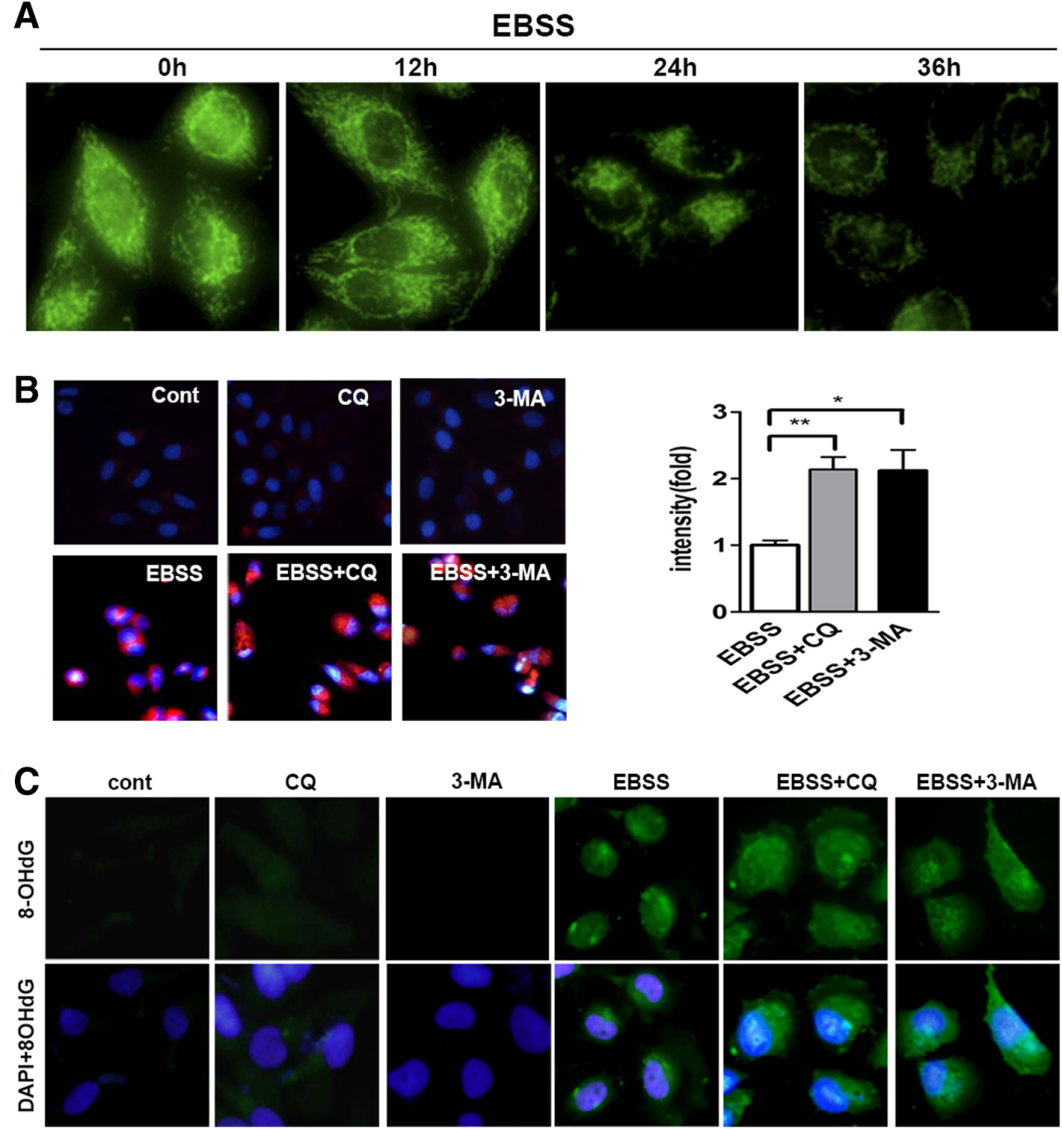
**Autophagy inhibition resulted in a loop of increasing mitochondrial ROS accumulation under nutrient deprivation.** (**A**) Mitochondrial membrane potential was examined by Rho123 under a fluorescence microscope after EBSS treatment of indicated times. Representative photos of cells were taken a magnification × 400. (**B**) After indicated treatments for 24 h, Chang liver cells were incubated with MitoSOX Red (red staining) DAPI dye (blue staining). Representative images of cells were taken a magnification × 400 (left). Relative fluorescence intensity of MitoSOX Red per cell for each condition was quantitated in columns. Data were shown as mean ± SEM (n = 3; *: p < 0.05; **: p < 0.01). (**C**) After indicated treatments for 24 h, oxidative damaged DNA was visualized by immunocytochemistry for 8-OHdG (green) and counter stained with DAPI (blue) at a magnification × 400.

Damaged mitochondria are the main source of ROS [14], so we next examined the mitochondria quality of Chang liver cells in the nutrient-free condition. Damaged mitochondria were correlated with low mitochondrial membrane potential which could be detected by Rho123 [15]. Our results showed that Rho123 fluorescence intensity continuously decreased in a time-dependent manner under nutrition deprivation (Figure 6A). Then mitochondrial ROS was examined by MitoSOX™ Red, which is a special mitochondrial superoxide indicator. In the nutrition-free condition, hang liver cells emitted strong red fluorescence, which was further increased by co-treatment with CQ or 3-MA (Figure 6B). These results suggested that under nutrient deprivation, abundant ROS were released by damaged mitochondria, which could be further aggravated when autophagy was inhibited.

ROS lead to DNA oxidative damage, which could be assayed by 8-Hydroxy-2’deoxy Guanosine (8-OHdG) staining. Massive immunoreactivity of 8-OHdG was found in EBSS-treated cells, and much stronger fluorescence intensity was observed after co-treatment with CQ or 3-MA (Figure 6C). Most notably, fluorescence staining for 8-OHdG mostly localized in cell nuclei in the EBSS group, but a large part of 8-OHdG localized in cytoplasm in the cells of the EBSS + CQ group. Since only mitochondrial DNA (mtDNA) localizes in cytoplasm but not nucleus, the increased oxidative damaged DNAs were very likely to be mtDNA. Damaged mtDNA is also an indicator of mitochondria damage [14]. Therefore, these results suggested that in the nutrient-free condition, autophagy reduced the generation of mitochondrial ROS and thus prevented further mitochondria damage which otherwise would produce more ROS in the cells. Therefore, when autophagy is inhibited, there might have a loop that would increase mitochondria damage and ROS accumulation, finally very likely leading to the increase of cell necrosis under nutrient deprivation.

**Figure 7:**
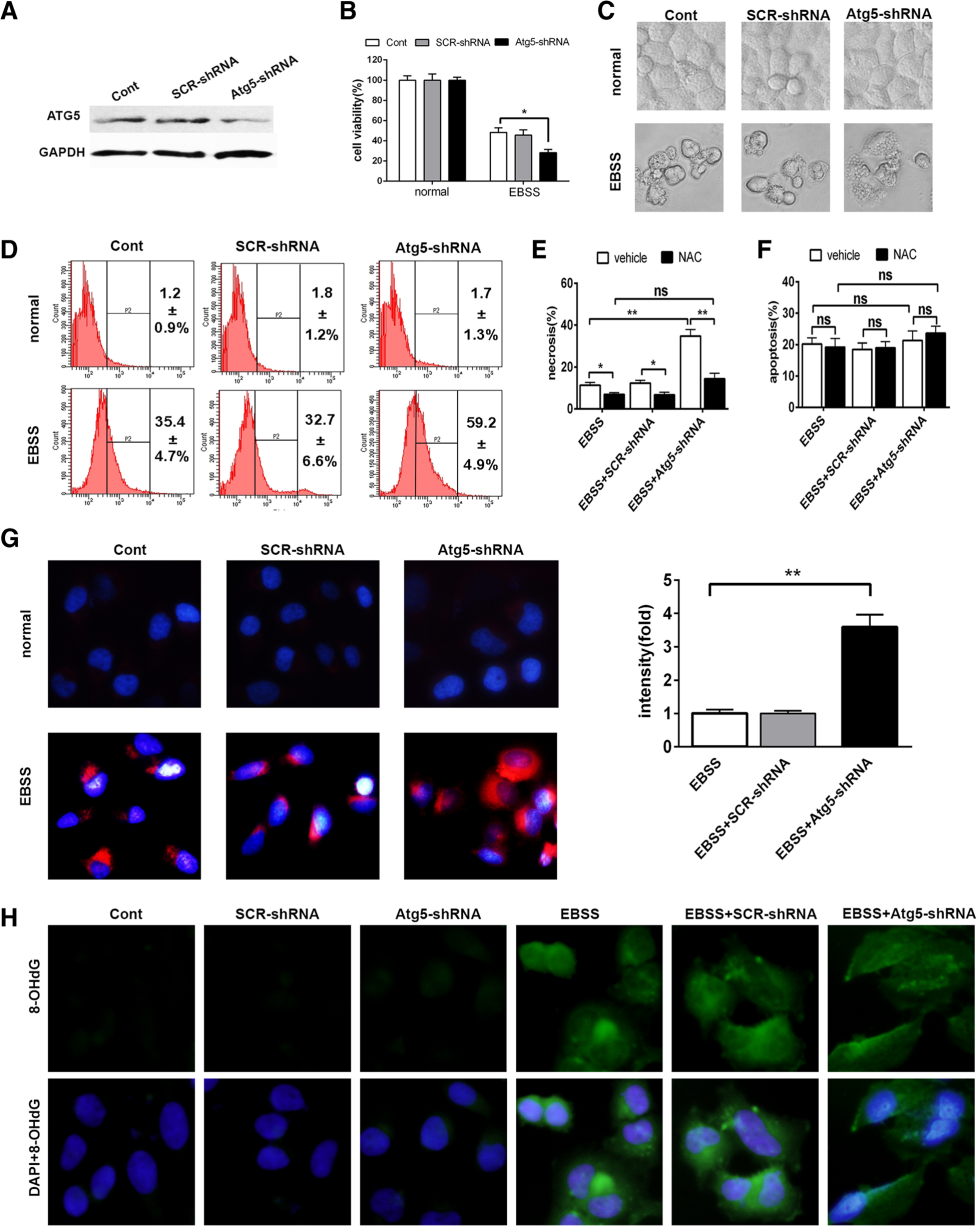
**Atg5 deficiency aggravated EBSS-induced mitochondrial ROS generation of liver cells and subsequent cell necrosis.** Chang liver cells were transfected with the indicated lentiviruses. **(A)** Atg5 expression of the transfected cells were determined by immunblotting. (**B-H**) The transfected cells were cultured with complete medium or EBSS for 24 h. (**B**) Cell viability was measured by CCK8. Data were shown as mean ± SEM (n = 6; *: p < 0.05). (**C**) The morphologies of cells were captured by a light microscope. (**D**) ROS generation was measured by flow cytometry using DCF-DA staining. ROS positive cells were counted and expressed as the mean ± SEM (n = 3). (**E** and **F**) The percentage of necrotic (**E**) and apoptotic (**F**) cells were assayed by Hoechst 33342/PI staining. Data were shown as mean ± SEM (n = 3; *: p < 0.05; **: p < 0.01; ns: no significance). (**G**) The cells were incubated with MitoSOX Red (red staining) and DAPI dye (blue staining). Representative images of cells were taken a magnification × 400 (left). Relative fluorescence intensity of MitoSOX Red per cell was quantitated and shown as mean ± SEM (n = 3; **: p < 0.01). (**H**) Oxidative damaged DNA was visualized by immunocytochemistry for 8-OHdG (green) and counter stained with DAPI (blue) at a magnification × 400.

To further confirm the role of autophagy in the liver cells under nutrient deprivation, we silenced the expression of the essential autophagy gene Atg5 in the Chang liver cells by lentivirus-delivered shRNA (Figure 7A). Under nutrient-free condition, Atg5-shRNA group has less cell viability compared to control and SCR-shRNA groups (Figure 7B), and displayed the morphological features of necrotic cell (Figure 7C). Flow cytometry assay showed that Atg5-shRNA group had higher percentage of ROS-generating (DCF positive) cells than control and SCR-shRNA groups 24 h after EBSS treatment (Figure 7D). Antioxidant NAC remarkably reduced EBSS-induced cell necrosis but not apoptosis (Figure 7E and F). And Atg5 deficiency had no significant impact on EBSS-induced cell necrosis after NAC treatment (Figure 7E). Continuing studies showed that under nutrient deprivation condition, Atg5 deficiency led to more mitochondrial ROS production and oxidative damage of mitochondria (Figure 7G and H). These data further suggested that autophagy prevented liver cells from nutrient-free induced necrosis by eliminating ROS-generating mitochondria.

### Antioxidant protects autophagy inhibition induced liver injury under ischemia *in vivo*

**Figure 8:**
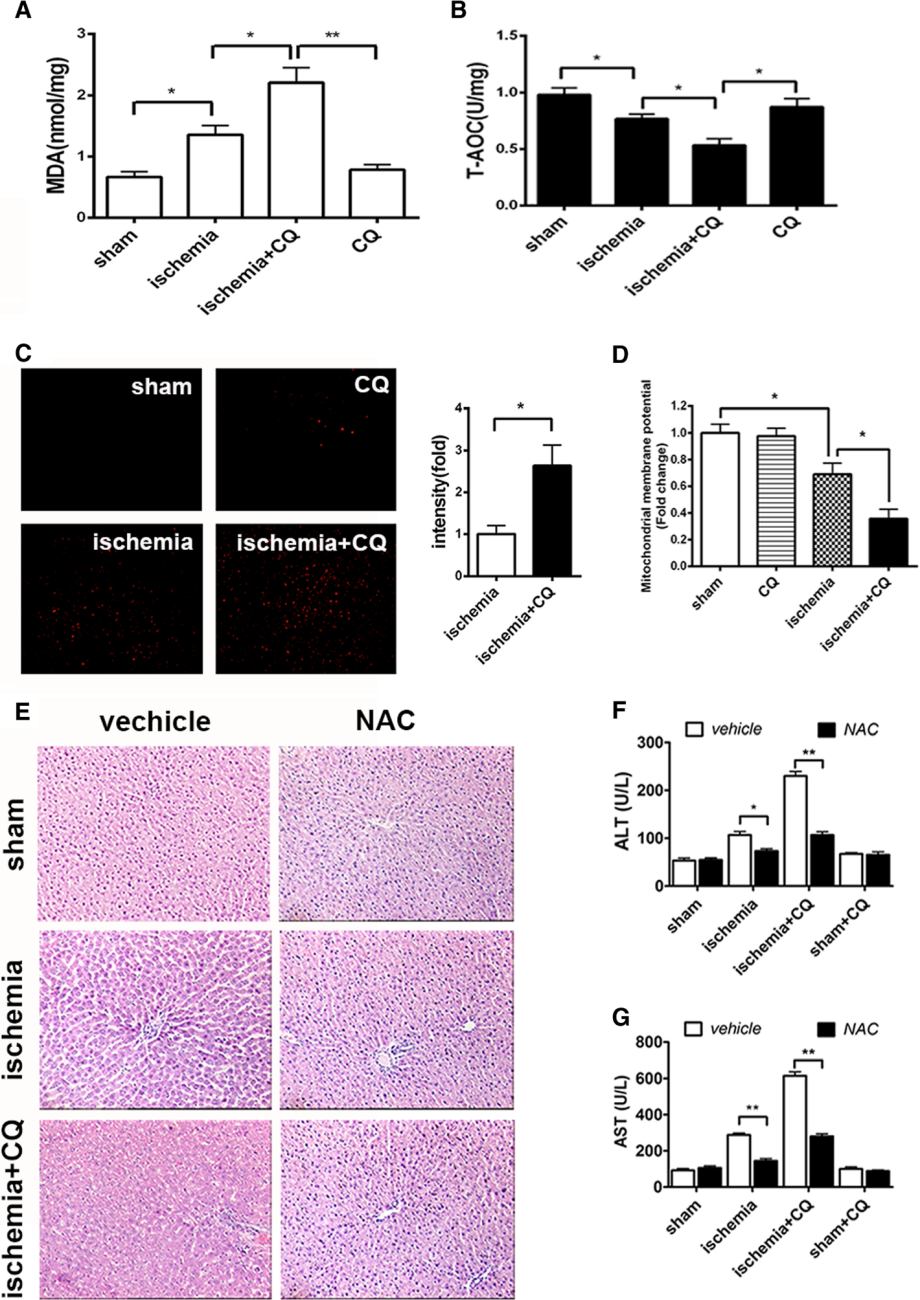
**Antioxidant attenuated autophagy inhibition induced aggravation of ischemic liver injury *in vivo*.** The rats were treated as indicated. (**A** and **B**) Levels of MDA (**A**) and T-AOC (**B**) of rat livers were detected. Data were shown as mean ± SEM (n = 6; *: p < 0.05; **: p < 0.01). (**C**) MitoSOX Red staining positive cells of liver cryosections were identified by fluorescence microscopy (left panel) and quantified (right panel). Data were shown as mean ± SEM (n = 3; * p < 0.05). (**D**) Mitochondrial membrane potential of rat livers were detected by Rho123 staining and analyzed by FACScan flowcytometry. Data were shown as mean ± SEM (n = 3; * p < 0.05). (**E**) Images were representative H&E staining of the liver sections with an original magnification × 200. (**F** and **G**) Serum levels of ALT (**F**) and AST (**G**) of rats were detected. Data were shown as mean ± SEM (n = 6; *: p < 0.05; **: p < 0.01).

We next determined whether the protective mechanism of autophagy in nutrient deprivation also worked *in vivo*. Firstly, we examined whether autophagy inhibition resulted in the increase of oxidative stress in the ischemic liver by detecting the content of malondialdehyde (MDA), which is used to assess lipid peroxidation, and total anti-oxidation competence (T-AOC). The results showed that ischemia gave rise to the upregulation of MDA level and the downregulation of T-AOC level, and this trend was further exacerbated by co-treatment of CQ (Figure 8A and B). Further study showed that ischemia led to vast generation of mitochondrial ROS in the rat liver, and co-treatment of CQ aggravated this ischemia-induced ROS production (Figure 8C). Meanwhile, the mitochondrial membrane potential of rat liver cells was determined by flow cytometry analysis of Rho123 staining. The results suggested that CQ enhanced the ischemia-induced decline of mitochondrial membrane potential of rat liver cells (Figure 8D). These data revealed that autophagy inhibition aggravated ischemia-induced mitochondrial oxidative stress.

Pathological structure analysis showed that NAC treatment prominently reduced the hepatic structural damage in the ischemia and ischemia + CQ groups. And there was no obvious histological difference between the ischemia + NAC and ischemia + CQ + NAC groups (Figure 8E). Consistent with these results, pretreatment with NAC significantly reduced the elevated levels of serum ALT and AST in the ischemia + CQ group (Figure 8F and G). These results clearly indicated that autophagy played its protective role by suppressing ischemia-induced mitochondrial ROS accumulation.

## Discussion

Using a series of morphological and biochemical assays, we found that autophagy was activated in the process of ischemia, and moderated ischemia-induced liver injury. In order to explore the underlying mechanism, the major damage factor of ischemia, nutrient deprivation, was mimicked through EBSS treatment in Chang liver cells. Inhibition of starvation-induced autophagy significantly increased cell necrosis but not apoptosis *in vitro*. Further studies showed that autophagy inhibition aggravated starvation-induced ROS accumulation, especially mitochondrial ROS, and mitochondria damage. The increase of starvation-induced cell necrosis and ischemia-induced liver damage, both of which resulted from autophagy inhibition, could be reduced by antioxidant NAC.

Our results showed mitochondria of cells deprived of nutrition were disrupted, and produced abundant ROS (Figure 6A, B and Figure 7G). ROS can induce oxidative damage of organisms, macromolecules, including DNA, lipids and proteins [16-18]. Excessive ROS could result in cell necrosis [19]. Part of the damaged mitochondria were normally sequestered and degraded through autophagy, which helped cells to escape from cell death [13]. But when autophagy was inhibited, this process was hampered and thereby leading to damaged mitochondria accumulation, subsequently to more ROS production, and ultimately to more cell necrosis. It was a “vicious cycle” in which initial ROS-induced mitochondria damage enhanced ROS production that, in turn, led to further mitochondrial damage and eventually massive hepatocytes death.

On the basis of the results above, we concluded that during the process of ischemia, autophagy generally was induced to decrease cell necrosis and liver injury mainly through suppressing ROS accumulation, especially produced by mitochondria. However, patients usually had underlying diseases including metabolic syndrome, diabetes, hypertension, and advanced age. Many of these conditions have been shown to interfere with autophagy [20]. And various studies showed that livers with impaired autophagy were vulnerable to hepatic I/R [21,22]. Wang JH *et al*. found that livers of older patients had significantly less reparative capacity following I/R injury, which occurred during these operations. Immunoblot, autophagic flux, genetic, and imaging analyses all showed that autophagy inhibition increased the sensitivity of liver to I/R injury. Atg4B overexpression blocked the mitochondrial permeability transition and decreased cell death induced by I/R in old patients [21]. In addition, another recent study showed that autophagic proteolysis was inhibited in steatotic liver, due to impairment of autophagosome acidification and cathepsin expression. Using a murine model, Takeshi Suzuki et al. provided evidence that the steatotic liver was vulnerable to hepatic I/R [23]. And in the study of Ramalho FS *et al*. steatotic livers showed impaired regenerative response and reduced tolerance to hepatic injury compared with non-steatotic livers [24]. All evidences above showed that inhibition of autophagy was correlated with high sensitivity of liver to injury. However, these studies did not investigate that which stage of I/R, ischemia or reperfusion, is the main stage in which autophagy exerts protective effect. Or maybe autophagy protects liver in the whole process of I/R? In this report, we showed that autophagy, at least, was an important protector in the process of ischemia, although the exact role of autophagy in reperfusion needs further investigation.

Restoration or enhancement of autophagy may ameliorate the damage to liver function in the process of ischemia, especially to livers with low level of autophagy. Further work about this will provide more applicable therapeutic strategy about the pretreatment of liver surgery.

## Materials and methods

### Animals and experimental design

Male Sprague–Dawley rats (10–12 week-old, weighing 220–250 g) were obtained from the Shanghai Experimental Center, Chinese Science Academy, Shanghai, and were maintained at an animal facility under pathogen-free conditions. The animals were housed in a temperature and humidity controlled environment with a 12 h light/12 h dark cycle. All animals received humane care according to animal protocols approved by the Second Military Medical University Animal Care Committee.

48 rats were randomly divided into eight equal groups, including sham, ischemia, sham + CQ, sham + NAC, sham + CQ + NAC, ischemia + CQ, ischemia + NAC, ischemia + CQ + NAC groups. All animals were fasting overnight before operation. CQ and NAC (both from Sigma-Aldrich, St Louis, MO) were used as autophagy inhibitor and antioxidant, respectively. CQ (60 mg/kg) and NAC (150 mg/kg) were given by intraperitoneal injection to rats 2 hours before sham or ischemia operation.

### Surgical procedure

Rats were anaesthetized by sodium phenobarbital at a dose of 30 mg/kg. A complete midline incision was made. Hepatoduodenal ligament was separated after entry into the belly. The hepatic pedicle including hepatic artery and portal vein, which supplies the left and median liver lobes (70% of liver mass), was occluded with a microvascular clamp for 90 min [25]. Sham-operated rats were only subjected to anesthesia without ischemia operation. Then livers and bloods of rats were immediately collected without reperfusion.

### Biochemical analysis

Serum ALT and AST were analyzed using a Fuji DRICHEM 55500 V (Fuji Medical System, Tokyo, Japan) according to the manufacturer’s instructions. The levels of MDA and T-AOC were measured using the assay kits (Nanjing Jiancheng Bioengineering Institute, Nanjing, China), according to the manufacturer’s instructions.

### Cell culture

Human liver cell line Chang liver was maintained in RMPI1640 medium (GIBCO, Invitrogen, Carlsbad, CA) supplemented with 10% fetal bovine serum (GIBCO), 100 units/ml penicillin, and 100 μg/ml streptomycin in a humidified incubator under 95% air and 5% CO_2_ at 37°C.

### Nutrient deprivation and drug treatments of cells

To obtain nutrient-free condition, hepatocytes were washed three times with phosphate buffered saline (PBS) and incubated in EBSS (Sigma-Aldrich, E2888) for indicated time at 37°C. CQ, 3-MA (Sigma-Aldrich) and NAC were used at 10 μM, 5 mM and 1 mM for indicated time, respectively.

### Transient transfection and identification of autophagy

GFP-tagged LC3 expression vector was utilized to demonstrate the occurrence of autophagy. Chang liver cells were seeded (7 × 10^3^ cells/well) in 96-well plates and cultured overnight, then GFP-LC3 expression plasmids were transiently transfected into the cells using Fugene HD transfection reagent (Roche, Basle, Switzerland), according to the manufacturer’s instructions. The cells were subjected to the indicated treatments 24 h after transfection. At the end of the treatments, the puncta were observed under a fluorescent microscope (Olympus IX71, Olympus Optical Co. Ltd, Tokyo, Japan). A minimum of 200 cells per sample was counted in triplicate for each experiment.

### Gene silencing with lentivirus-delivered shRNA

shRNA candidate target sequence to Atg5 is 5’-CCTTTCATTCAGAAGCTGTTT-3’. Scrambled (SCR) shRNA sequence, which was used as a negative control, is 5’-TTCTCCGAACGTGTCACGT-3’. The oligonucleotides encoding the Atg5-shRNA or SCR-shRNA sequence were inserted into the GFP express vector pGCL-GFP (Shanghai GeneChem, shanghai, china). The recombinant virus was packaged using Lentivector Expression Systems (Shanghai GeneChem). Chang liver cells were infected. After 3 days, GFP-positive cells were counted under fluorescence microscope. Atg5 expression after shRNA infection was revealed by western blot analysis at 4th day.

### CCK8 assay

The measurement of viable cell mass was performed with CCK8 (Dojindo Laboratories Co., Kumamoto, Japan). Cells (7 × 10^3^ cells/well) were seeded in 96-well plates and cultured overnight, and then were treated as indicated. As soon as the treatments were completed, 10 μl solution of CCK8 was added to each well. These plates were continuously incubated for 1 h in a humidified CO_2_ incubator at 37°C. Finally, the absorbance of sample was measured on a microplate reader ELX800 (BIO-TEK Instruments, Inc, Winooski, VT) at 490 nm.

### Cell death analysis

The percentages of apoptotic or necrotic cells were assessed by Apoptosis and Necrosis Assay Kit (Beyotime, Haimen, Jiangsu, China). After incubation, cells were stained with Hoechst 33342 and PI and then examined by fluorescence microscopy. The apoptotic cell showed a high Hoechst 33342 staining and a low PI staining while its nucleus was condensed or fragmented. The PI strong positive and Hoechst weak positive cells were regarded as necrotic ones. In four microscopic fields containing 200 cells, the number of viable cells, necrotic cells and apoptotic cells was counted [26,27].

### Histological analysis, immunohistofluorescence and tunel staining

Collected livers were fixed with 10% neutral buffered formalin and embedded in paraffin. All paraffin-embedded sections were stained with H&E for conventional morphological evaluation. The primary immunohistofluorescence antibody is HGMB1 (Abcam, Cambridge, UK). Tunel staining (Calbiochem, La Jolla, CA) was used to assess the apoptosis level of paraffin-embedded fraction slides, according to the manufacturer’s instructions.

### Western blot analysis

Whole cell lysates were subjected to SDS–PAGE. The blots were incubated with desired primary antibodies, which included anti-LC3 (Novus Biologicals. Littleton, CO), anti-p62, anti-cleaved caspase3, anti-cleaved caspase7 and anti-Atg5 (all from Cell Signaling Technology, Beverly, MA), and then with anti-rabbit IgG peroxidase conjugated secondary antibody (Hangzhou HuaAn Biotech, Hangzhou, Zhejiang, China) and chemiluminescent substrates. Hybridization with anti-GAPDH (Hangzhou HuaAn Biotech) was used to confirm equal protein loading.

### Mitochondria isolation from liver

The mitochondria of rat livers were prepared using Tissue Mitochondria Isolation Kit (Beyotime), according to the manufacturer’s instructions.

### Measurement of intracellular ROS level and mitochondrial superoxide level

Cells were incubated with 10 μM 2’, 7’-dichlorofluorescein diacetate (DCF-DA) for 20 min at 37°C to assess intracellular ROS level. After washing twice in PBS, positively stained cells were observed under fluorescent microscope and quantified with Image J software (US National Institutes of Health, Bethesda, MD), or were analyzed at an excitation wavelength of 480 nm and an emission wavelength of 525 nm by BD FACScan flowcytometry (BD Biosciences, San Jose, CA).

To examine accumulation of mitochondrial superoxide, cells were incubated with 2.5 μM MitoSOX Red mitochondrial superoxide indicator (Invitrogen) for 10 min, and then were washed twice with PBS and fixed with 4% paraformaldehyde for 15 min. Cell nuclei were labeled using DAPI (1 mg/ml) for 4 min. Fluorescent images were captured using a fluorescence microscope. Quantification of MitoSOX Red fluorescence was also analyzed with excitation/emission at 510/580 nm using a FACScan flowcytometry (BD Biosciences).

Freshly prepared frozen liver sections were incubated with 2 μM MitoSOX™ Red mitochondrial superoxide indicator (Invitrogen) for 30 min at 37°C. Then they were observed by fluorescence microscopy and quantified with Image J software.

### Mitochondrial membrane potential examination

Mitochondrial membrane potential of chang liver cell was measured by the incorporation of a cationic fluorescent dye Rhodamine 123 (Rho123, 5 μg/ml, Sigma). After the indicated treatment periods, the cells were stained with Rho123 and incubated for 15 min at 37°C in the dark. The fluorescence intensity of cells was observed under fluorescence microscopy.

Freshly prepared mitochondrial suspensions (0.5 mg protein/ml) of rat livers were incubated with 2 mM Rho123 for 30 min 37°C in the dark and then washed and suspended in PBS. Samples were analyzed immediately with excitation/emission at 488/530 nm using a FACScan flowcytometry.

### Immunocytochemistry

Cells (4 × 10^4^ cells/well) were seeded in 24-well plates and cultured overnight. At the end of the designated treatments, cells were washed with PBS and fixed with 4% paraformaldehyde for 15 min. After incubation for 1 hour in blocking buffer (10% goat serum in PBS), the cells was incubated with anti-8-OHdG (Abcam) antibody at 4°C overnight. On the following day, cells were washed twice with PBS and incubated with second antibody at room temperature for 45 min. After washing with PBS, cell nuclei were stained using DAPI (1 mg/ml) for 4 min. Then cells were observed under fluorescent microscope.

### Statistical analysis

Data were presented as mean ± SEM. Differences were analyzed by the Student t test and one-way ANOVA. A p value of less than 0.05 was considered statistically significant. Statistical analysis was performed with GraphPad Prism 5.0 software (GraphPad Software, San Diego, CA).

## Abbreviations

I/R: Ischemia/reperfusion; CQ: Chloroquine; ROS: Reactive oxygen species; NAC: N-acetyl-L-cysteine; ALT: Alanine aminotransferase; AST: Aspartate aminotransferase; MDA: Malondialdehyde; T-AOC: Total anti-oxidation competence; H&E: Hematoxylin and eosin; 3-MA: 3-Methyladenine; EM: Electron microscope; EBSS: Earle’s balanced salt solution; LC3: Microtubule-associated protein 1 light chain 3; Rho123: Rhodamine 123; DCF-DA: 2’,7’-Dichlorofluorescein diacetate; 8-OHdG: 8-Hydroxy-2’-deoxy Guanosine; mtDNA: Mitochondrial DNA; HP: High power field.

## Competing interests

The authors declare that they have no competing interests.

## Authors’ contributions

KS and XQX contributed equally to this manuscript. KS, XQX, JRS and LXW conceived and designed the study. XQX, YL and ZPH performed animal experiments. KS and XZ performed cell experiments. KS, XQX and NC performed molecular experiments. KS, XQX and SSZ wrote the draft manuscript. LXW finalized the manuscript. All authors read and approve the final manuscript.
